# Functionalized Periodic Mesoporous Silica Nanoparticles for Inhibiting the Progression of Atherosclerosis by Targeting Low-Density Lipoprotein Cholesterol

**DOI:** 10.3390/pharmaceutics16010074

**Published:** 2024-01-04

**Authors:** Hao Jin, Wenbin Lu, Yahao Zhang, Yong Wu, Jiandong Ding, I. R. Chiara Villamil Orion, Cihui Liu

**Affiliations:** 1 Department of Cardiology, Zhongda Hospital Affiliated with Southeast University, Nanjing 210009, China; 230219025@seu.edu.cn (H.J.); 230229099@seu.edu.cn (Y.Z.); 230229501@seu.edu.cn (Y.W.); 223227044@seu.edu.cn (I.R.C.V.O.); 2 Department of Biomedical Sciences, Nanjing Normal University, Nanjing 210023, China; cihui@njnu.edu.cn

**Keywords:** functionalized periodic mesoporous silica nanoparticles, atherosclerosis, low-density lipoprotein cholesterol

## Abstract

Atherosclerotic disease is a substantial global burden, and existing treatments, such as statins, are recommended to lower low-density lipoprotein cholesterol (LDL-C) levels and inhibit the progression of atherosclerosis. However, side effects, including gastrointestinal unease, potential harm to the liver, and discomfort in the muscles, might be observed. In this study, we propose a novel method using periodic mesoporous silica nanoparticles (PMS) to create heparin-modified PMS (PMS-HP) with excellent biocompatibility, enabling selective removal of LDL-C from the blood. In vitro, through the introduction of PMS-HP into the plasma of mice, we observed that, compared to PMS alone, PMS-HP could selectively adsorb LDL-C while avoiding interference with valuable components such as plasma proteins and high-density lipoprotein cholesterol (HDL-C). Notably, further investigations revealed that the adsorption of LDL-C by PMS-HP could be well-fitted to quasi-first-order (R^2^ = 0.993) and quasi-second-order adsorption models (R^2^ = 0.998). Likewise, in vivo, intravenous injection of PMS-HP enabled targeted LDL-C adsorption (6.5 ± 0.73 vs. 8.6 ± 0.76 mM, *p* < 0.001) without affecting other plasma constituents, contributing to reducing intravascular plaque formation (3.66% ± 1.06% vs. 1.87% ± 0.79%, *p* < 0.05) on the aortic wall and inhibiting vascular remodeling (27.2% ± 6.55% vs. 38.3% ± 1.99%, *p* < 0.05). Compared to existing lipid adsorption techniques, PMS-HP exhibited superior biocompatibility and recyclability, rendering it valuable for both in vivo and in vitro applications.

## 1. Introduction

According to the World Health Organization (WHO), the global prevalence of atherosclerosis (AS) is estimated to have contributed to 17 million deaths annually, making it one of the leading chronic diseases worldwide. In the United States, AS emerged as a primary cause of cardiovascular diseases, such as heart failure (HF), myocardial infarction (MI), and stroke, resulting in 610,000 deaths from cardiovascular diseases each year [1]. Nowadays, it is widely accepted that elevated blood lipid concentrations, especially for LDL-C, are associated with an increased risk of AS and cardiovascular disease [2,3,4]. Therefore, targeting interventions aimed at reducing LDL-C levels have subsequently prevented serious cardiovascular events like MI, HF, and stroke, which ultimately reduce mortality rates.

In addition to healthy lifestyle interventions, patients with atherosclerotic disease derive benefits from various therapies, including statins, fibrates, niacin, ezetimibe, and bile acid sequestrants. However, it should be noted that lipid-lowering medications could also give rise to several side effects, including gastrointestinal discomfort, impaired liver function, new-onset diabetes, arrhythmias, and muscle pain [5,6].

There has been increased interest in adsorbents, such as resins, as researchers have discovered their efficacy in lipid adsorption and reducing blood lipid levels. Resin, however, exhibited poor biocompatibility, which represented a significant drawback [7,8].

Composed of silicon elements, periodic PMS has attracted significant attention in recent decades. Until now, PMS has been widely applied as a promising platform in the field of biomedicine via injection, owing to its good biocompatibility and biodegradability [9,10]. Similarly, due to these characteristics of PMS, composite systems based on PMS as a carrier have been extensively explored in the field of AS [11]. Song et al. based their work on PMS, employing hyaluronic acid modification and subsequently loading simvastatin to inhibit the progression of AS. The research demonstrated that this system was capable of reducing the secretion of pro-inflammatory cytokines TNF-α and IL-6 and preventing macrophage foam cell formation, thereby impeding the advancement of AS [12].

Interestingly, the characterization of PMS has demonstrated its potential as an adsorbent, which is attributed to its pore structure and large surface area. It exhibits excellent adsorption capabilities without inducing additional biological chemical reactions. [13,14]. More importantly, it was found that PMS successfully led to a significant reduction in body weight and fat composition in animals that received PMS alongside a high-fat diet, as compared to mice treated without PMS [15]. However, it remains unknown whether PMS could prevent the progression of AS via injection, as this adsorption was nonspecific.

Previous research has found that heparin is a natural sulfated glycosaminoglycan that can selectively bind to the positively charged ApoB-100 protein on the surface of LDL-C and demonstrates excellent hemocompatibility [16].

**Figure 1:**
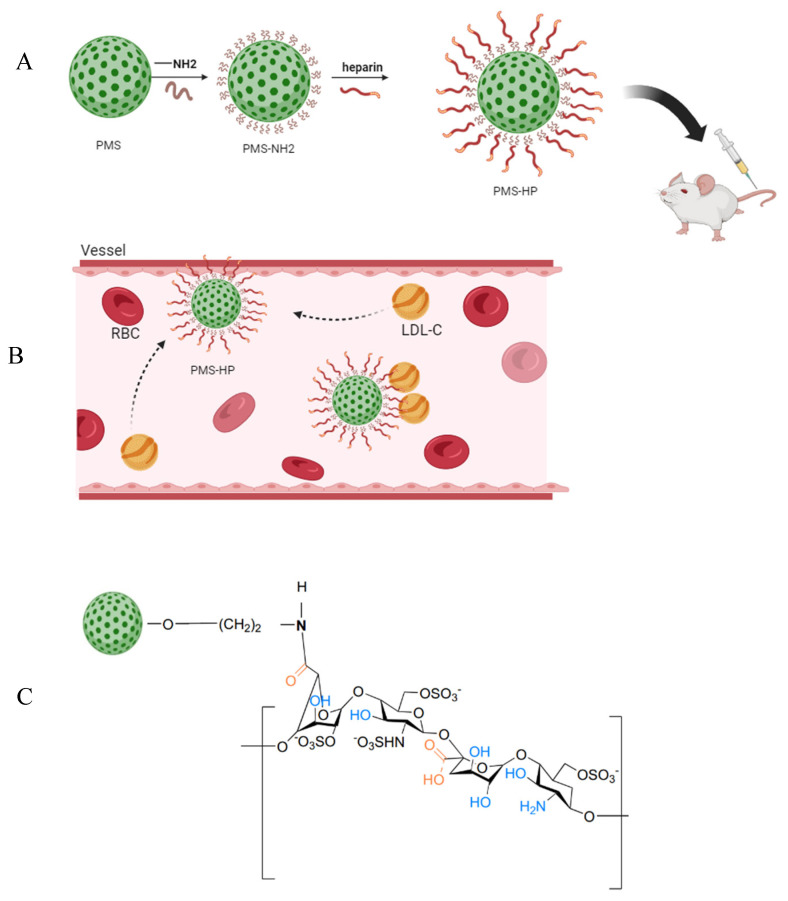
Model diagram of preparing PMS-HP for targeting LDL-C. (**A**) Schematic diagram of the fabrication process of PMS-HP; (**B**) PMS-HP contributing to targeting and adsorbing LDL-C; (**C**) Molecular structure of PMS-HP.

Therefore, our study postulated that PMS modified with heparin could achieve selective adsorption of LDL-C in the bloodstream when administered intravascularly, hindering the progression of AS (Figure 1A,B).

## 2. Materials and Methods

### 2.1. Materials

Absolute ethanol, toluidine blue (TB), hexyl hydride, hydrochloric acid (HCl), and NaCl were obtained from the Institute of Cardiovascular Diseases, Southeast University. Oil Red O staining solution (G1015-100 mL), polyformaldehyde, hematoxylin, eosin staining kit, and phosphate-buffered saline (PBS) were purchased from Servicebio (Wuhan, China). The Masson’s staining kit (G1340-100 mL) was purchased from Solarboi Life Sciences (Beijing, China). 1-ethyl-3-(3-dimethylaminopropyl) carbodiimide hydrochloride (EDC), N-hydroxy succinimide (NHS), 2-(N-morpholino) ethanesulfonic Acid (MES) were purchased from Aladdin Bio-Chem Technology Co (Shanghai, China). Standard concentrations of LDL-C (1 mmol/L) and HDL-C (1 mmol/L) were purchased from Nanjing Jiancheng Bioengineering Institute (Nanjing, China). Sigma-Aldrich (Steinheim, Germany) provided cetyltrimethylammonium chloride (CTAC), triethanolamine (TEA), and tetraethyl orthosilicate (TEOS). (3-aminopropyl) triethoxysilane (APTES) was purchased from Macklin. Co., Ltd. (Shanghai, China). Heparin sodium was obtained from Tianjin Biochemical Pharmaceutical Co., Ltd. (Tianjin, China). Cy5-heparin was purchased from QIYUE BIOLOGY (Xi’an, China).

### 2.2. Preparation of PMS-HP

#### 2.2.1. Preparation of PMS

The synthesis of PMS was carried out as follows: Initially, 8 g CTAC and 0.068 g TEA were successively dissolved in 20 mL of water at 95 °C with vigorous stirring. After 1 h, a gradual addition of 1.5 mL of TEOS took place, and the resulting mixture underwent an additional hour of stirring. The resultant products were retrieved through centrifugation and subjected to multiple washes with ethanol to eliminate any residual reactants. Subsequently, these collected products were subjected to a 12-h extraction process involving a mixture of ethanol and hydrochloric acid at 60 °C to eliminate the CTAC template, a process that was repeated several times. Eventually, any remaining surfactants and organic materials were entirely removed through calcination at 450 °C. The resulting material was then dispersed in water after undergoing ultrasonic treatment and three rounds of centrifugal cleaning [17]. In our study, the production of PMS from the reagents yielded approximately 45% of the theoretical yield.

#### 2.2.2. Preparation of PMS-NH2

Amino-functionalized nanoparticles (PMS-NH2) were prepared through a modified approach, as outlined in the reference [18]. A total of 340 mg of PMS was suspended in ethanol, and 4.1 mmol of APTES was carefully added dropwise. The reaction was allowed to progress at 40 °C overnight. Subsequently, the nanoparticles were separated by centrifugation (12,000 rpm for 20 min), thoroughly washed with ethanol, and designated as PMS-NH2. Compared to PMS, there was almost no loss, with a PMS-NH2 yield of approximately 100%.

#### 2.2.3. Preparation of PMS-HP

The general synthesis procedure for PMS-HP was as follows: MSN-NH2 was uniformly dispersed in a 30 mL solution of 0.1 M MES. Subsequently, 287.4 mg of EDC, 172.8 mg of NHS, and 2 mg of heparin were added. The mixture was then shaken overnight at 4 °C on a shaker. Afterward, a solid product was obtained by centrifugation. The molecular structure of PMS-HP is shown in Figure 1C. Similarly, PMS-HP-Cy5 was also prepared with HP-Cy5 for future use. Compared to PMS-NH2, there was almost no loss, with a PMS-HP yield of approximately 100%.

### 2.3. Characterization

#### 2.3.1. Morphology Characterization by TEM

The morphology and porous structure were verified using transmission electron microscopy (TEM) (JEM-2100; JEOL, Tokyo, Japan) with an accelerating voltage of 120.00 kV. Prior to examination, the samples were well dispersed in water through sonication and deposited on carbon-coated copper grids immediately. The grids were subsequently dried overnight at ambient temperature.

#### 2.3.2. Zeta Potential and DLS Characterization

The surface electrical potential (Zeta potential) was measured using a Zetasizer Nano-ZS ZEN3600 (Malvern Instruments, Worcestershire, UK) at 25 °C with the nanoparticles dispersed in water at room temperature with an applied field strength of 20 V/cm. The dynamic light scattering (DLS) was determined using the same equipment. All samples for zeta potential and DLS were prepared by ultrasonic dispersing.

#### 2.3.3. XPS Characterization

XPS measurements were conducted using an ESCALAB 250Xi spectrometer (Thermo Scientific, Waltham, MA, USA) equipped with a pass energy of 30 eV and a power of 100 W (operating at 10 kV and 10 mA) alongside a monochromatized AlKα X-ray source (hν = 1486.65 eV). All sample analyses were performed at a pressure below 1.0 × 10^−9^ Pa. Spectra were acquired using the Advantage software (Version 5.979) with an increment step of 0.05 eV.

#### 2.3.4. Quantification of Amin Groups for PMS-NH2

The amino group content was determined by the following procedure: 0.5 g of PMS- NH2 was added to a 10 mL 0.01 M HCl standard solution. The mixture was stirred thoroughly at room temperature and allowed to react for 5 h. Afterward, centrifugation was performed, separating the carrier particles from the supernatant, which were then placed into separate beakers.

The carrier particles were washed multiple times with deionized water, and the washing solution was combined with the previously collected supernatant. The combined solution was titrated using a 0.01 M NaOH solution until the solution’s color changed from colorless to pink. The volume of NaOH solution V consumed during titration was used to calculate the amino group content [19].

$$Content\;ofamino\;groups=\frac{\left(10.0-V\right)}{0.5}*0.01$$

#### 2.3.5. Quantification of Heparin in PMS-HP [20]

(1) Preparation of Toluidine Blue Solution

A total of 1.0 g of NaCl, 0.025 g of TB, and 0.53 g of 36% HCl were dissolved in a clean beaker. After dissolution, the solution was transferred to a 500 mL volumetric flask using a glass rod. The beaker was washed with distilled water twice, and the washing liquid was collected into the volumetric flask. Finally, the volume was adjusted, the solution was well-shaken, and it was stored at room temperature in a brown wide-mouth bottle.

The concentrations of the prepared solution were TB 0.005%, NaCl 0.2%, and HCl 0.01 mol/L.

(2) Preparation of Heparin Solution

Preparation of standard calibration solution of heparin sodium (0.5 mg/mL).

(3) Establishment of Standard Curve

First, 7 centrifuge tubes labeled 1 to 7 were taken, and 0.5 mL of the TB solution was added to each tube. Subsequently, 0, 40, 80, 100, 160, 200, and 240 μL of heparin sodium solution and 500, 460, 420, 400, 340, 300, and 260 μL of water were added, respectively. Then, the tubes were vortexed for 30 s, followed by the addition of 1 mL of hexyl hydride to each tube (Table 1). Using a vortex mixer, vigorous vortexing for 30 s extracted the complex formed between toluidine blue and heparin into the organic phase. The layers were allowed to separate, the lower aqueous phase was collected, and absorbance at 631 nm was measured on a microplate reader within 30 min. The measurement data were processed, and a standard curve was plotted.

(4) Quantitative Analysis of Polymer Surface Heparin

EP tubes were taken, and 12.5 mg of PMS-HP was added. The tubes were then filled with 500 μL of water, followed by the addition of 0.5 mL of TB solution. Vortexing for 30 s at room temperature was performed using a vortex mixer. Subsequently, 1 mL of hexyl hydride was added to each tube, and vigorous vortexing for 30 s was carried out. After settling, the phases separated into an upper organic phase, a middle aqueous phase, and a lower solid phase. The middle aqueous phase was collected into a 96-well plate, and absorbance at 631 nm was measured using a microplate reader.

#### 2.3.6. The Stability of PMS-HP and the Release of HP

Five 50 mL centrifuge tubes were prepared, and each was filled with 50 mL of PBS and 500 mg of PMS-HP. These tubes were placed in a constant temperature shaker set at 37 °C with a speed of 120 rpm/min. They were then shaken for 0, 4, 8, and 24 h, respectively, before being centrifuged at 12,000 rpm for 10 min. The resulting supernatant and precipitate were obtained, and the same detection method (Section 2.3.5) was employed to measure the content of HP in both the supernatant and the precipitate (PMS-HP-1) [21].

### 2.4. The Capacity of PMS-HP Compared to PMS for Adsorbing LDL-C or HDL-C in the Solutions of Different Concentrations

First, 10, 20, 40, 60, 80, and 100 μL of a standard LDL-C solution (1 mM) was introduced into six separate 1.5 mL centrifuge tubes. To each of these, incremental volumes of water (90, 80, 60, 40, 20, and 0 μL) were added accordingly. The final concentrations of the LDL-C solution were 0.1, 0.2, 0.4, 0.6, 0.8, and 1.0 mM. Subsequently, 1 mg of PMS was incorporated into each solution, which was then shaken at 4 °C for a duration of 1 h. After the incubation period, the solutions underwent centrifugation at 12,000 rpm and 4 °C for 10 min. The resulting supernatant was carefully collected for quantifying LDL-C concentration. The sediment, comprising PMS-LDL-C, was subjected to two washes with water to eliminate impurities. Subsequently, the PMS’s capacity to adsorb LDL-C was calculated by comparing the LDL-C volume present in the supernatant with that in the initial solutions [22].

Similarly, various concentrations of HDL-C were prepared utilizing a standard HDL-C solution (1 mM). In a parallel manner, the supernatants from these HDL-C solutions were collected to determine HDL-C concentration. The sediment, composed of PMS-HDL-C, was also obtained and subjected to the same washing process. The capacity of PMS to adsorb HDL-C was subsequently computed.

Likewise, the capacity of PMS-HP to adsorb LDL-C or HDL-C was similarly calculated using analogous procedures, and the PMS-HP-LDL and PMS-HP-HDL were acquired.

In addition, different particles acquired were characterized with TEM and ZetaSizer Nano.

### 2.5. Kinetic Study of the Adsorption Process

In this experiment, we investigated the kinetic characteristics of PMS-HP adsorbing LDL-C by varying the adsorption time. LDL-C solution with a concentration of 0.5 mM was prepared. For each of the six 1.5 mL centrifuge tubes, 100 μL of the solution was added, followed by the addition of 1 mg PMS-HP. The mixtures were then shaken for 0.5, 1.0, 1.5, 2.0, 2.5, and 3.0 h to complete the adsorption process. Subsequently, the adsorption capacity of PMS-HP on LDL-C at different time points was determined.

Moreover, fitting analysis was conducted on the adsorption using the pseudo-first-order adsorption kinetic equation and the pseudo-second-order adsorption kinetic equation. The pseudo-first-order adsorption kinetic equation (Equation (1)) and the pseudo-second-order adsorption kinetic equation (Equation (2)) were as follows [23]:

(1) ln⁡(qe−qt)=lnqe−k1t

(2) $$\frac{t}{qt}=\frac{1}{k2qe}+\frac{t}{qe}$$

In the equations, *qe* represents the saturated adsorption capacity of PMS-HP adsorbing LDL-C; *qt* represents the amount of LDL-C that was adsorbed by PMS-HP at time *t*; *k*1 and *k*2 are the adsorption constants in the pseudo-first-order and pseudo-second-order adsorption kinetic equations, respectively.

### 2.6. Toxicity of PMS-HP to HUVECs

Human umbilical vein endothelial cells (HUVECs) were purchased from Procell Life Science&Technology Co., Ltd. (Wuhan, China). A total of 1 × 10^4^ HUVECs were seeded into each well of a 96-well plate and cultured in the cell culture incubator for 24 h. Then, different concentrations of PMS-HP (0–100 µ/mL^−1^) were added to the 96-well plate and co-incubated for 24 h. Afterward, the viability of the cells was determined using a CCK-8 reagent. During the experiment, the wells without cells served as a blank group. The control group of cells was treated with 0 µg/mL^−1^ of PMS-HP.

$$Cell\;viability(\%)=\frac{ODtest-ODblank}{ODcontrol-ODblank}*100\%$$

### 2.7. Animals

Six-week-old male ApoE^−/−^ mice (SPF) were obtained from Cavens Co., Ltd. (Nanjing, China) and housed in a specific pathogen-free facility. The mice were housed under controlled conditions of 55% relative humidity, 22 °C room temperature, and a 12-h cycle of light and dark. The animal study protocol was reviewed and approved by the Southeast University Animal Welfare Committee. After one week of acclimation with a standard chow diet and water, the ApoE^−/−^ mice were fed an atherogenic high-fat/cholesterol diet for an 8-week feeding period to establish AS mice [24]. The AS mice would be used for further experiments.

### 2.8. The Ability of PMS-HP Compared to PMS for Selectively Adsorbing LDL-C in Plasma

Plasma was extracted from the AS mice (N = 8) and heated to 80 °C for 1.5 min to eliminate lipases, thereby safeguarding LDL-C and HDL-C from hydrolysis.

Considering the limited availability of plasma per mouse, 100 μL of plasma was obtained from each of the 4 mice and subsequently diluted with PBS to a total volume of 300 μL for subsequent use.

Subsequently, 300 μL of diluted plasma from each mouse was individually added to three separate 1.5 mL centrifuge tubes (100 µL each). Subsequently, 1 mg of either PMS or PMS-HP was introduced into the respective tubes, while the remaining tube served as the control.

The tubes were then shaken at room temperature for 1 h. After subjecting the mixture to centrifugation at 12,000 rpm for 10 min, the resulting supernatant was collected to quantify the concentrations of LDL-C, HDL-C, and total protein (TP). The concentrations were measured with a full-automatic biochemical analyzer (Chemray800, Rayto, Shenzhen, China).

### 2.9. Metabolism of PMS-HP In Vivo

#### 2.9.1. Pharmacokinetic and Biodistribution of PMS-HP in Blood

To conduct a pharmacokinetic and biodistribution study, AS mice (N = 4) received intravenous administration of PMS-HP (labeled with Cy5) (25 µg/g). Blood circulating profiles were assessed by collecting blood samples (30 µL) at various intervals (1 min, 1 h, 2 h, 3 h, 4 h, 6 h, 12 h, and 24 h post-injection) via cheek pouch puncture. Fluorescent imaging was conducted on the blood samples using an IVIS imaging system (PerkinElmer, Inc., Waltham, MA, USA) with excitation at 650 nm and emission at 670 nm. Data analysis was performed using Living Image software (version 4.4). To normalize for individual variability, data at each time point were calculated relative to the initial time point (1 min post-injection).

Exploration of PMS-HP distribution in major organs (heart, liver, spleen, lung, kidney, and brain) involved harvesting these organs at different intervals post-injection (12 h, 48 h, 7 d, and 14 d). Ex vivo imaging of the harvested organs was conducted using IVIS, and subsequent data processing was performed using Living Image software.

#### 2.9.2. Excretion of PMS-HP

Our PMS particles had a size of over 100 nm, making renal excretion unlikely. Previous studies indicated that PMS could be excreted through the intestines and detected in the feces of mice. The potential mechanism involved entry into the intestine through bile excretion and relied on detecting the presence of Si with ICP-OES to confirm the existence of PMS in feces [25,26].

In our study, AS mice (N = 4) were administered PMS-HP intravenously (25 µg /g). Stool samples (1 g) were collected from the mice before PMS-HP injection and at 1, 2, 3, 7, and 14 days post-injection. Subsequently, the silicon content in the stool was measured using the iCE™ 3400 AAS atomic absorption spectrometer. The steps for detecting the silicon content can be summarized as follows: Digestion and release of the silicon element were achieved by adding an appropriate amount of stool samples (1 g) into nitric acid. Then, the response signal values of the silicon element in the samples were measured using the atomic absorption spectrometer.

### 2.10. Blood Lipid and TP in Mice Treated with PBS or PMS-HP In Vivo

AS mice were randomly divided into two groups. The control group (N = 4) received PBS treatment through the tail vein, while the experimental group (N = 4) received PMS-HP treatment (25 μg/g/2 weeks) via the tail vein. All mice underwent PMS-HP or PBS treatment for 2 months.

Subsequently, blood samples were collected at two weeks and two months post-injection. These samples were then centrifuged at 3000 rpm for 30 min to separate the plasma. The plasma samples were analyzed using a fully automated biochemical analyzer (Chemray800, Rayto, China) to measure the levels of LDL-C, HDL-C, and TP.

### 2.11. Plaque on the Aortic Wall and in the Tricuspid Valve of Mice Treated with PBS or PMS-HP In Vivo

After being treated with PBS or PMS-HP for 2 months (25 μg/g/2 weeks), AS mice (N = 4) were anesthetized thoroughly and sacrificed. Following perfusion with saline, the aorta was longitudinally dissected from the ascending aorta to both common iliac arteries. After staining the aorta with Oil Red O for 30 min, 60% isopropanol was used to decolorize the tissue. Using Image J, the surface areas of the plaques and the overall arterial wall were measured after photographs were taken. In our study, we evaluated the severity of AS by using the ratio of plaque area to total vessel area.

Furthermore, to evaluate atherosclerotic lesions in the aortic root, consecutive sections (8 μm) were cut through the region where the valve cusp was visible, followed by staining with Oil Red O and decolorizing with isopropanol. The detectable areas of atherosclerotic lesions were expressed as a percentage of the total vessel wall area [27].

### 2.12. Fibrosis in the Tricuspid Valve of the Heart in Mice Treated with PBS or PMS-HP In Vivo

Similarly, after AS mice were treated with PMS-HP or PBS for 2 months (25 μg/g/2 weeks), AS mice (N = 4) were euthanized and sacrificed. The hearts were perfused with saline and preserved in 10% paraformaldehyde at 4 °C. To assess the extent of remodeling and fibrosis, consecutive sections (8 μm) were sliced through the region of the aortic sinus where the valve cusp was visible, followed by staining with Masson’s trichrome. In our study, we assessed the severity of vascular fibrosis by using the ratio of cross-sectional fibrotic area to total cross-sectional vessel area.

The steps for staining with Masson’s trichrome can be summarized as follows: The tissue sections were dehydrated using xylene and ethanol, followed by staining with hematoxylin for 10 min. After rinsing and differentiation with hydrochloric acid, a combined staining with Masson’s trichrome was performed for 10 min. Subsequently, the sections were treated with 1% phosphomolybdic acid solution, aniline blue solution, and xylene for processing. Finally, the staining process was completed [27].

### 2.13. Safety Evaluation of PMS-HP

#### 2.13.1. Toxicity of PMS-HP in Mice

Through the tail vein, PMS-HP (25 μg/g/2 weeks) was injected into mice (N = 4), and 30 μL of peripheral blood was collected from the mice before injection and at 0.5, 2, 24, 72 h, 7 days, and 14 days after PMS-HP injection, which was used to measure blood parameters, including aspartate aminotransferase (AST), alanine aminotransferase (ALT), creatinine (Cr), blood urea nitrogen (BUN), white blood cells (WBC), red blood cell (RBC), hemoglobin (HGB), and plate (PLT).

In addition, in our study, the major organ toxicity of PMS-HP was assessed at 1 month and 2 months post-injection. Mice (N = 4) were administered PBS for 2 months and PMS for 1 month or 2 months (25 μg/g/2 weeks). Then, the major organs, including the heart, liver, spleen, lung, kidney, and brain, were collected and processed into paraffin sections for hematoxylin-eosin staining (HE).

#### 2.13.2. Red Blood Cell Hemolysis Test

A total of 200 µL of blood was collected from mice and centrifuged at 1000 rpm for 5 min to separate the red blood cells (RBCs). The RBCs were then washed five times with normal saline (NS) and resuspended in 4 mL of NS. Next, three 1.5 mL centrifuge tubes were prepared, each containing 900 µL of the erythrocyte suspension. To one tube, 100 µL of NS (negative control group) was added, while to another tube, 100 µL of water (positive control group) was added. In the third tube, 100 µL of PMS-HP dissolved in NS solution was added. The tubes were incubated at 37 °C in a water bath for 2 h. Following incubation, the mixture was centrifuged at 1000 rpm for 10 min, and the degree of hemolysis was visually evaluated. Additionally, 100 µL of the supernatant was collected from each tube to measure the absorbance at 570 nm. The formula for hemolysis is:

$$Hemolysis\;(\%)=\frac{ODexperiment-ODnegative}{ODpositive-ODnegative}$$

In addition, the RBCs treated with different treatments, including NS, water, and PMS-HP, were also observed with SEM [28].

### 2.14. Recovery and Recycling of PMS-HP

A total of 1 mg of PMS-HP was introduced into a 1.5 mL centrifuge tube, and 100 μL (0.5 mM) of LDL-C solution was added. Subsequently, the mixture was shaken for 1 h.

Following this, the mixture was centrifuged at 12,000 rpm at 4 °C for 10 min. The resulting supernatant was collected to quantify the amount of LDL-C adsorbed with PMS-HP, while the sediment contained PMS-HP-LDL-C.

The PMS-HP-LDL-C sediment was then subjected to washing with a high-concentration NaCl solution to isolate the PMS-HP.

Subsequently, the recovered PMS-HP was employed for the adsorption of LDL-C in consecutive rounds for the second, third, and fourth times.

(1) Quantification of the recovered PMS-HP

We obtained recovered PMS-HP that had undergone 4 cycles. Subsequently, following the aforementioned testing method (Section 2.9.2), nitric acid was added, and the relative silicon element content was measured with an iCE™ 3400 AAS atomic absorption spectrometer to indirectly assess the changes in PMS-HP. We compared the variations in recovered PMS-HP under different cycle numbers.

(2) Quantification of HP in the recovered PMS-HP

We obtained recovered PMS-HP that had undergone 4 cycles. Subsequently, employing the same testing method (Section 2.3.5) as described earlier, we assessed the surface HP content of recovered PMS-HP at different cycle numbers.

(3) The recyclable PMS-HP for adsorbing LDL-C

Comparing the changes in LDL-C levels in the solution before and after treatment with recovered PMS-HP, we evaluated the removal efficiency of LDL-C with recovered PMS-HP.

### 2.15. Statistical Analysis

Continuous variables were described using mean ± standard deviation or interquartile range, while categorical variables were presented as percentages. Statistical analysis involved t-tests, ANOVA, or Mann–Whitney U tests for continuous data and chi-square analysis for categorical data. The significance levels were indicated as “NS” for no significant difference between groups, “*”, “**”, and “***” for significance at *p* < 0.05, *p* < 0.01, and *p* < 0.001, respectively. SPSS Statistics 26.0 (IBM, USA) was used for statistical analysis, and GraphPad Prism 7.0 (GraphPad Software, San Diego, CA, USA) was employed for graphical analysis.

## 3. Result

### 3.1. Characterization

**Figure 2:**
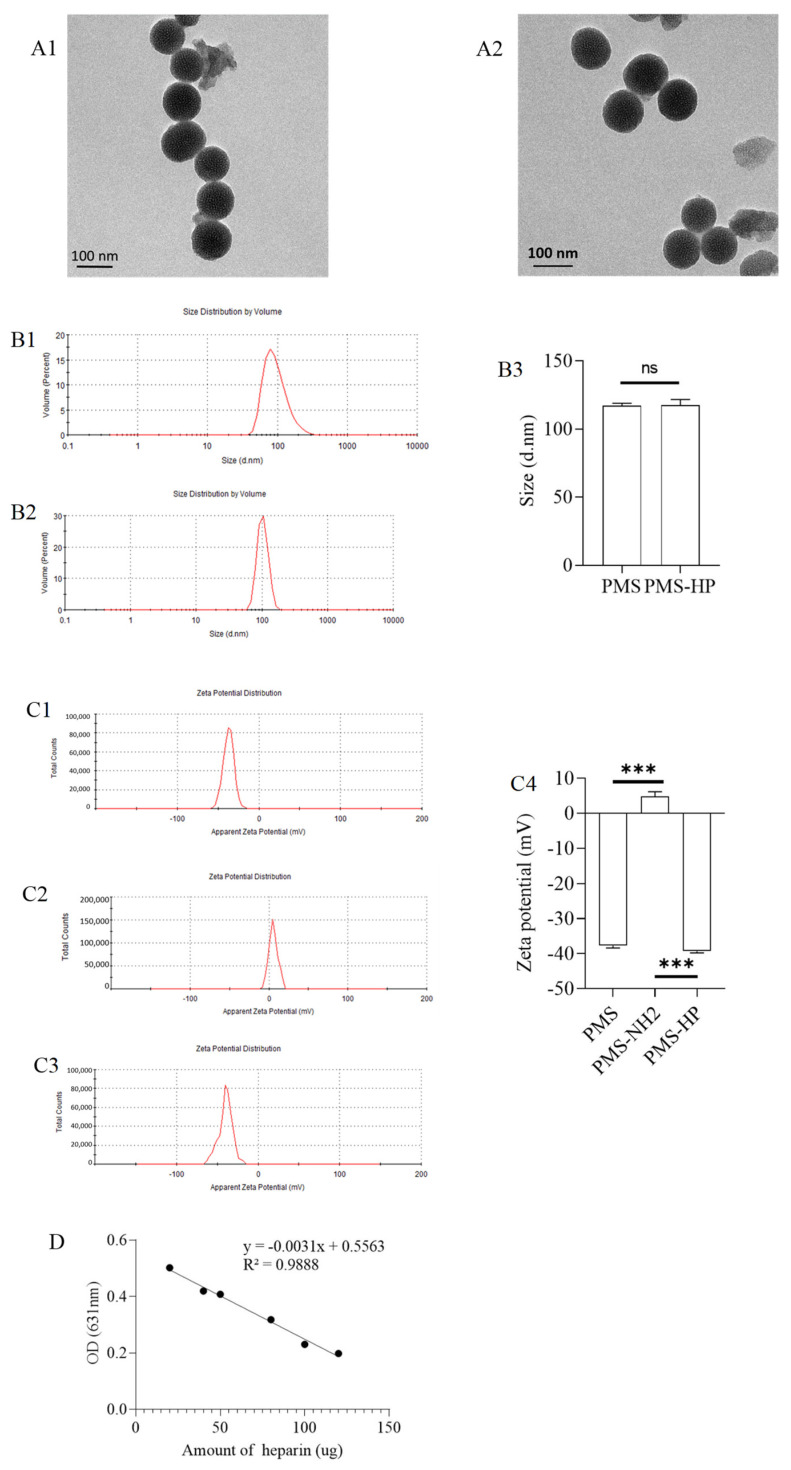

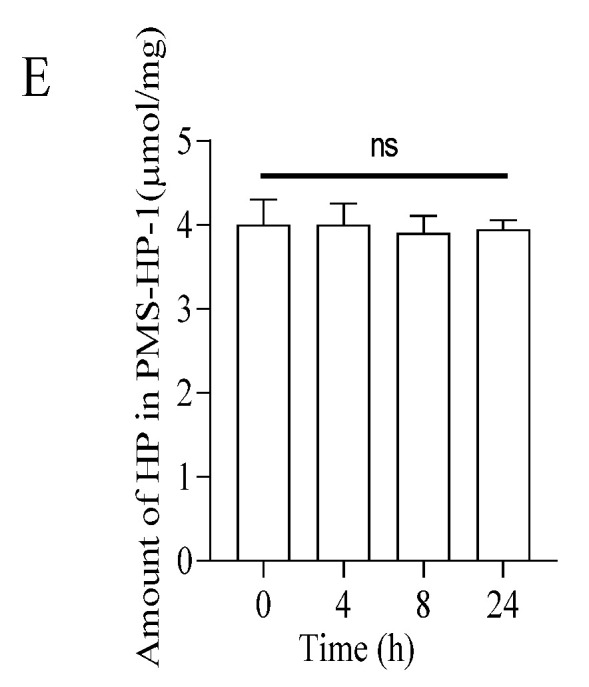
Characterization of PMS and PMS-HP. (**A1**) TEM images of PMS; (**A2**) TEM images of PMS-HP; (**B1**) Size of PMS; (**B2**) Size of PMS-HP; (**B3**) Comparisons of the size among the different particles (n = 3, P = 0.868); (**C1**) Zeta potential of PMS; (**C2**) Zeta potential of PMS-NH2; (**C3**) Zeta potential of PMS-HP; (**C4**) Comparisons of the zeta potential among the different particles (n = 3, P1 = 0.000, P1 = 0.000); (**D**) Standard curve for the quantitative analysis of heparin using toluidine blue; (**E**) Amount of HP on PMS-HP treated with PBS for 0, 4, 8 and 24 h (n = 3) (*** *p* < 0.001).

As shown in Figure 2(A1,A2), the PMS and PMS-HP exhibited excellent dispersion and a regular shape in the TEM images.

Furthermore, size measurements indicated that the PMS and PMS-HP had an approximate diameter of 100 nm (Figure 2(B1,B2)). The comparison of the size between PMS and PMS-HP showed no statistical significance (Figure 2(B3)).

In addition, the PMS’s surface potential had an initial negative charge (Figure 2(C1)). After amination modification, it shifted to a positive charge (Figure 2(C2)). Ultimately, following heparinization modification, it reverted to a negative charge again (Figure 2(C3)). The comparison was statistically significant (Figure 2(C4)), which demonstrated the successful heparin modification of PMS.

A total of 1 mg PMS-NH2 was modified with 0.32 µmol amin groups.

The standard curve for the quantitative analysis of heparin using toluidine blue is shown in Figure 2D. Moreover, 1 mg PMS-HP was modified with 3.99 µg heparin.

As depicted in Figure 2E, with the prolonged incubation and agitation time, within 24 h, there was no observable decrease in HP on the surface of the precipitate (PMS-HP-1), indicating the excellent stability of PMS-HP. Furthermore, upon further examination of HP content in the supernatant, similarly, within 24 h, no significant heparin release was detected.

### 3.2. In Vitro Studies

#### 3.2.1. The Ability of PMS-HP Compared to PMS for Adsorbing LDL-C or HDL-C In Vitro

**Figure 3:**
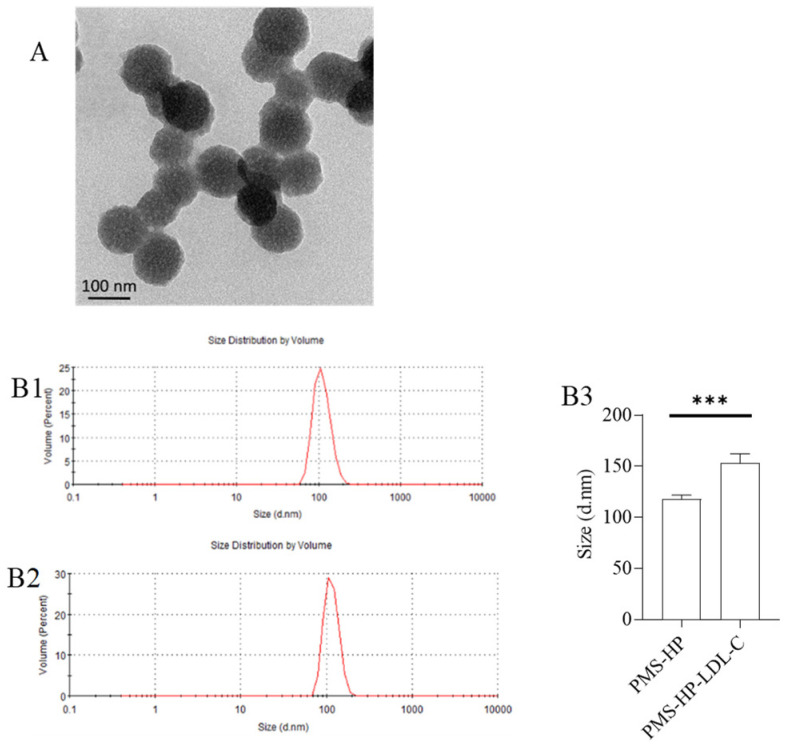
Characterization of PMS-HP after adsorbing LDL-C. (**A**) TEM image of PMS-HP after adsorbing LDL-C; (**B**) Size of PMS-HP before (**B1**) and after (**B2**) adsorbing LDL-C; (**B3**) Comparisons of the size of PMS-HP before and after adsorbing LDL-C (n = 3, *p* = 0.004) (*** *p* < 0.001).

Upon co-incubation of PMS-HP with LDL-C for a certain period, TEM observation (Figure 3A) revealed irregular shape and distribution of PMS-HP-LDL-C, indicating the adsorption of LDL-C. Furthermore, in comparison to PMS-HP (Figure 3(B1)), the PMS-HP-LDL-C complexes (Figure 3(B2)) showed a significant increase in particle size, and this difference was statistically significant (Figure 3(B3)), indicating successful adsorption of LDL-C.

**Figure 4:**
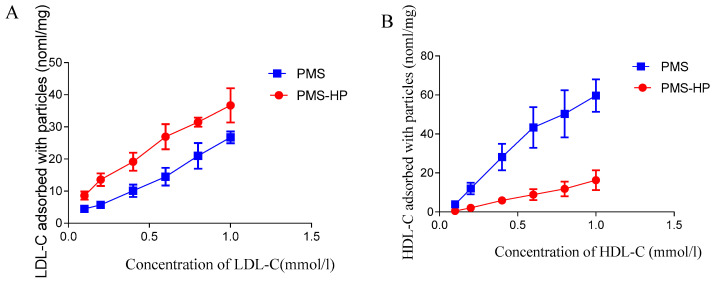
The capacity of PMS and PMS-HP for adsorbing LDL-C or HDL-C. (**A**) The capacity of PMS and PMS-HP for adsorbing LDL-C at different concentrations; (**B**) The capacity of PMS and PMS-HP for adsorbing HDL-C at different concentrations (n = 3).

To assess the adsorption capability of PMS and PMS-HP on LDL-C, varying concentrations of LDL-C were employed. As illustrated in Figure 4A, in contrast to LDL-C solutions with lower concentrations, both PMS and PMS-HP demonstrated enhanced effectiveness in adsorbing more LDL-C with higher concentrations. Notably, PMS-HP exhibited a greater capacity for adsorbing LDL-C in comparison to PMS.

Similarly, in the case of HDL-C, PMS exhibited heightened adsorption capabilities with higher concentrations. Remarkably, PMS exhibited greater adsorption of HDL-C, whereas PMS-HP displayed minimal HDL-C adsorption (Figure 4B).

In other words, compared to PMS, PMS-HP has the ability to adsorb a greater amount of LDL-C while having minimal impact on HDL-C levels.

#### 3.2.2. Kinetic Study of the Adsorption Process

**Figure 5:**
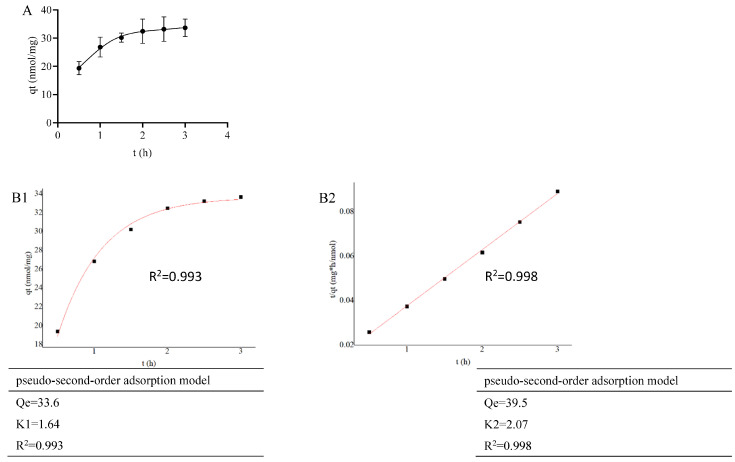
Adsorption kinetics of PMS-HP for adsorbing LDL-C. (**A**) The capacity of PMS-HP for adsorbing LDL-C at different times; (**B1**) Fitting result of pseudo-first-order adsorption model for PMS-HP adsorbing LDL-C; (**B2**) Fitting result of pseudo-second-order adsorption model for PMS-HP adsorbing LDL-C (n = 3).

For a fixed concentration of LDL-C solution, as the adsorption time increased, the adsorption of LDL-C by PMS-HP gradually intensified, eventually reaching a state of equilibrium (Figure 5A).

As shown in Figure 5(B1) (R^2^ = 0.993) and 5B2 (R^2^ = 0.998), PMS-HP exhibited good fitting to both first-order and second-order adsorption models for LDL-C in the adsorption kinetics fitting.

#### 3.2.3. Toxicity of PMS-HP to HUVEC

**Figure 6:**
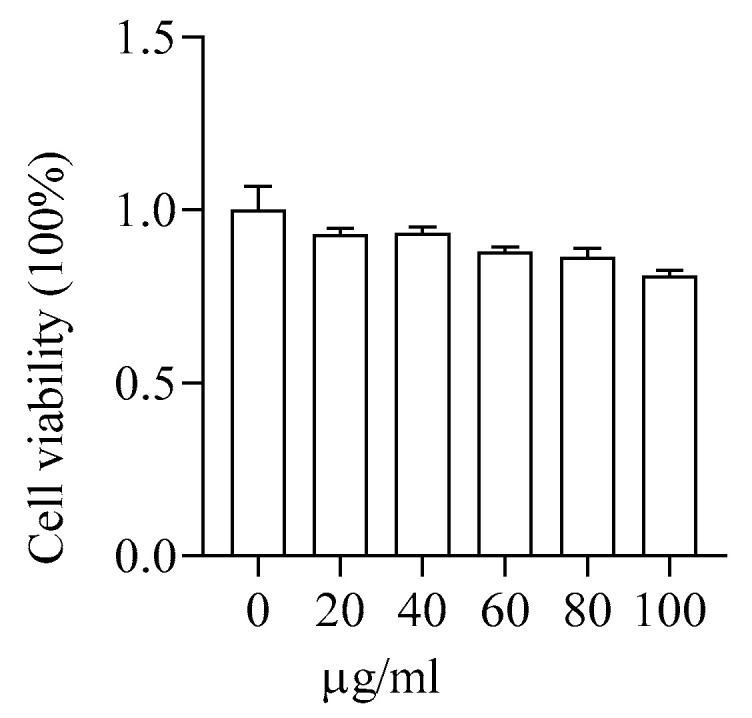
Safety assessment of PMS-HP in HUVECs. The cell viability of HUVECs was incubated with varying concentrations (0, 20, 40, 60, 80, 100 μg/mL) of PMS-HP for 24 h (n = 3).

The viability of HUVECs was assessed using CCK-8 reagent following incubation with various concentrations of PMS for 24 h. As depicted in Figure 6, PMS-HP did not induce notable toxicity, even at higher concentrations (100 µg/mL).

### 3.3. In Vivo Studies

#### 3.3.1. PMS-HP Selectively Adsorbing LDL-C from Plasma of AS Mice

**Figure 7:**
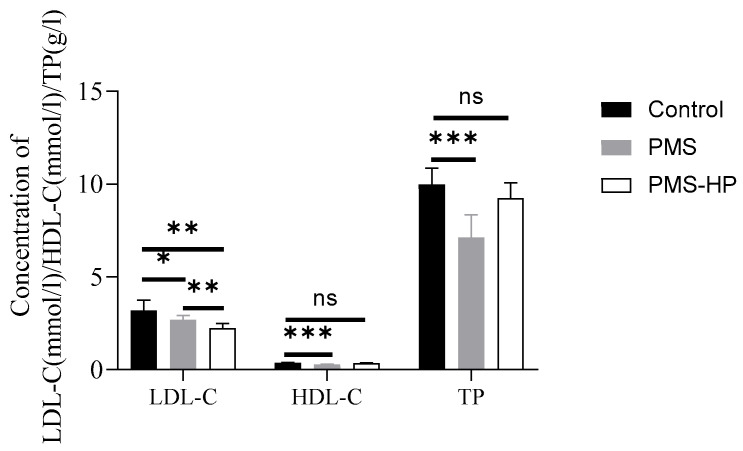
PMS-HP selectively adsorbed LDL-C from mice plasma in vitro in comparison to PMS. PMS-HP could efficiently adsorb LDL-C from plasma with minimal impact on HDL-C and TP levels. In contrast, PMS adsorption was non-selective, leading to the reduction of both HDL-C and TP in mice plasma (n = 8, P1 = 0.044, P2 = 0.001, P3 = 0.002, P4 = 0.000, P5 = 0.181, P6 = 0.000, P7 = 0.113; from left to right) (* *p* < 0.05, ** *p* < 0.01, *** *p* < 0.001).

With the plasma obtained from the AS mice and co-incubated with PMS-HP, PMS-HP demonstrated the ability to significantly reduce LDL-C levels in plasma while having minimal impact on HDL-C and TP levels. In comparison, although PMS effectively lowered LDL-C levels in plasma, it similarly affected HDL-C and TP levels. Importantly, PMS-HP exhibited a greater capacity for reducing LDL-C levels when compared to PMS (Figure 7).

#### 3.3.2. Metabolism of PMS-HP

##### Pharmacokinetic and Biodistribution of PMS-HP in Blood

**Figure 8:**
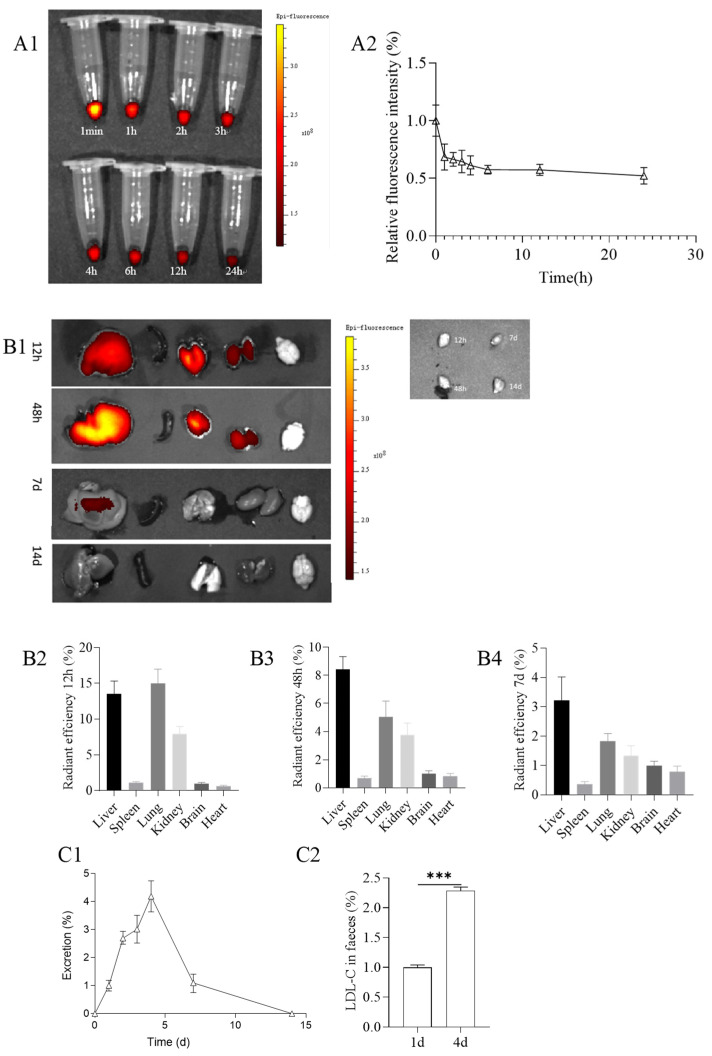
(**A1**) Metabolism of PMS-HP (labeled with Cy5) in blood at 1 min, 1, 2, 3, 4, 6, 12, and 24 h after the PMS-HP injection; (**A2**) Relative fluorescence intensity of PMS-HP-Cy5 in blood at 1 min, 1, 2, 3, 4, 6, 12, and 24 h after the PMS-HP injection; (**B1**) Representative ex vivo images of major organs (liver, spleen, lung, kidney, brain, and heart) at different time points (12 h, 48 h, 7 d, 14 d) after intravenous injection of PMS-HP-Cy5; Quantification of nanoparticles accumulation in major organs (liver, spleen, brain, lung, kidney) in comparison to brain at different time points ((**B2**) 12 h; (**B3**) 48 h; (**B4**) 7 d) after intravenous injection of nanoparticles; (**C1**) Excretion of PMS-HP at different time points (0 d, 1 d, 2 d, 3 d, 4 d, 7 d, and 14 d) in comparison to 1 d; (**C2**) Excretion of LDL-C at 4 d in comparison to 1 d (n = 4, *p* = 0.000) (*** *p* < 0.001).

With a solution of PMS-HP injected via the tail vein into the mice, blood samples were collected at different time points. As depicted in Figure 8(A1), PMS-HP was detectable in the bloodstream within 24 h after injection. Compared to the baseline concentration at 1 min post-injection, it was observed that PMS-HP exhibited favorable circulation time in the bloodstream (Figure 8(A2)).

With respect to the distribution of PMS-HP, after 12 h of injection, the nanoparticles were primarily distributed in the liver, lungs, and kidneys, with a higher concentration in the lungs. Subsequently, at 48 h after injection, the nanoparticles were still mainly distributed in the liver, lungs, and kidneys but with a predominant presence in the liver. After 7 days, there was a reduced distribution of nanoparticles, with a small residual presence in the liver. By 14 days, PMS-HP-Cy5 was not observed. (Figure 8(B1)).

Furthermore, to confirm that PMS-HP did not pass through the blood–brain barrier (BBB), we collected brain tissue for further examination of PMS-HP content. Using the aforementioned method (Section 2.9.2), we obtained 1 g of brain tissue, added nitric acid for digestion, and, upon testing, found no presence of silicon elements with the iCE™ 3400 AAS atomic absorption spectrometer. This confirmed that PMS-HP did not breach the BBB to enter the brain. Taking into account that the brain did not contain nanoparticles, we used brain fluorescence as a reference to quantitatively compare the fluorescence intensity in different organs. The results were consistent with the aforementioned findings, revealing the distribution of nanoparticles at 12 h (Figure 8(B2)), 48 h (Figure 8(B3)), and 7 days (Figure 8(B4)).

##### Excretion of PMS-HP

Regarding nanoparticle excretion, we used the excretion on the 1st day as a reference. It was observed that the highest excretion of nanoparticles occurred on the 4th day, with minimal excretion by the 14th day (Figure 8(C1)).

Furthermore, considering that PMS-HP was excreted from mice feces, we further assessed the content of LDL-C in the feces. Feces of mice from the first and fourth days (100 mg each) were separately mixed with 1 ml of concentrated saline to promote the release of LDL-C adsorbed with PMS-HP. The resulting suspension was then centrifuged at 3000 rpm for 30 min to obtain the supernatant, which was subsequently analyzed using a biochemical analyzer to determine the LDL-C content. We used the LDL-C content from the first day as the reference group and calculated the LDL-C content on the fourth day. As shown in the results, the excretion of LDL-C from mice on the fourth day was significantly higher than on the first day, consistent with the higher excretion of PMS-HP on the fourth day. It is evident that the LDL-C adsorbed by PMS-HP was also excreted from the feces (Figure 8(C2)).

#### 3.3.3. PMS-HP Selectively Adsorbing LDL-C from Plasma in AS Mice

**Figure 9:**
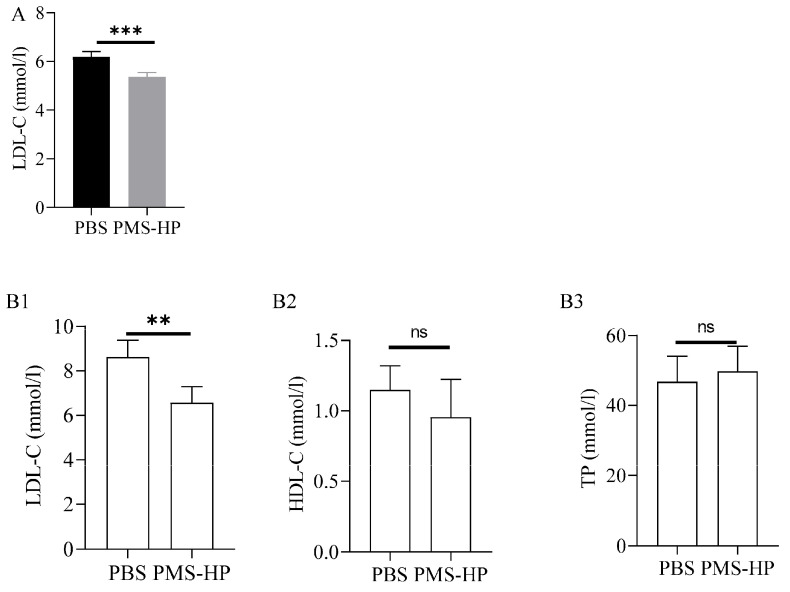
LDL-C,HDL-C, and TP levels of mice treated with PBS or PMS-HP. (**A**) LDL-C level of mice treated with PBS or PMS-HP for 2 weeks (n = 4, *p* = 0.001); (**B1**) LDL-C level of mice treated with PBS or PMS-HP for 2 months (n = 4, *p* = 0.008); (**B2**) HDL-C level of mice treated with PBS or PMS-HP for 2 months (n = 4, *p* = 0.27); (**B3**) TP level of mice treated with PBS or PMS-HP for 2 months (n = 4, *p* = 0.583) (** *p* < 0.01, *** *p* < 0.001).

As shown in the results, it can be observed that nanoparticle injection approximately reduced LDL-C by 0.8 mmol/L after administration in two weeks (Figure 9A).

After 2 months, in comparison to the mice treated with PBS, mice treated with PMS-HP showed a significant decrease in LDL-C (6.5 ± 0.73 vs. 8.6 ± 0.76 mM, *p* < 0.001) (Figure 9(B1)). More importantly, no significant difference was detected between the two groups with respect to HDL-C (0.95 ± 0.27 vs. 1.15 ± 0.17 mM, *p* > 0.05) (Figure 9(B2)) and TP (49.7 ± 7.2 vs. 46.8 ± 7.3 g/L, *p* > 0.05) (Figure 9(B3)).

#### 3.3.4. PMS-HP Reducing Plaque on the Aortic Wall and in the Tricuspid Valve in Mice

**Figure 10:**
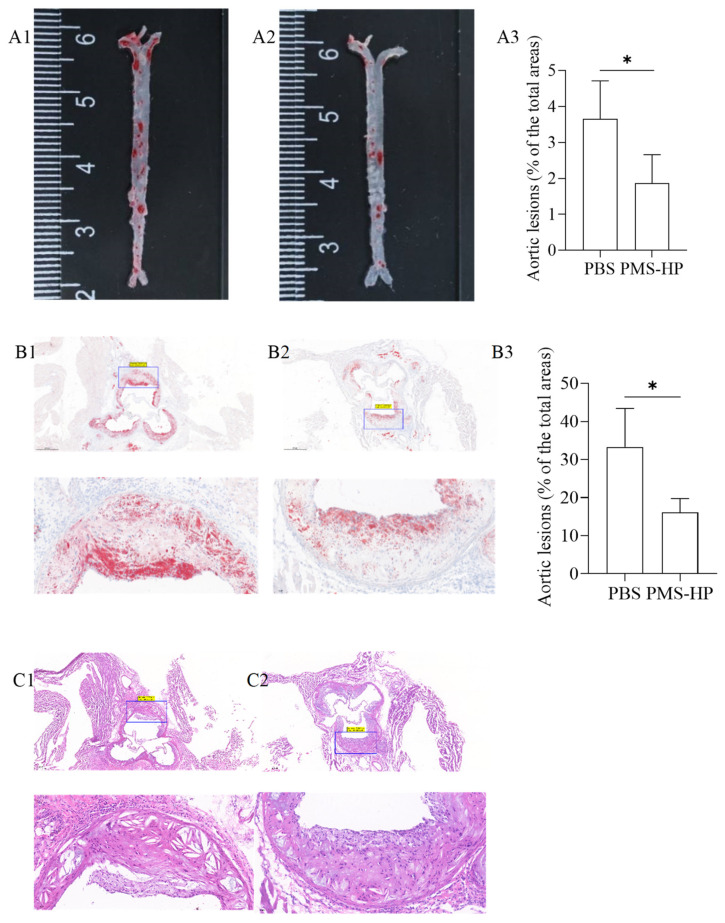
Representative photographs of Oil Red-O stained aortas and aortic roots from ApoE^−/−^after different treatments. Representative photographs of Oil Red-O stained aortas from mice treated with PBS (**A1**) and PMS-HP (**A2**); Comparisons of the Oil Red-O stained aortas between mice treated with PBS and PMS-HP (**A3**) (n = 4, *p* = 0.035); Representative photographs of Oil Red-O stained aortic roots from mice treated with PBS (**B1**) and PMS-HP (**B2**); Comparisons of the Oil Red-O stained aortic roots between mice treated with PBS and PMS-HP (**B3**) (n = 4, *p* = 0.019); Representative photographs of HE stained aortic roots from mice treated with PBS (**C1**) and PMS-HP (**C2**) (* *p* < 0.05).

After Oil Red-O staining, the aortic arch and abdominal aorta in the mice treated with PBS (Figure 10(A1)) displayed evident red-colored lipid deposits. Conversely, mice treated with PMS-HP (Figure 10(A2)) had a significant reduction in vascular plaques. The plaque percentage was calculated using Image-Pro-Plus 6.0 by dividing the plaque area by the total arterial area. As compared to the control group, the PMS-HP treatment group showed a significant reduction in the average percentage of atherosclerotic plaque area (3.66% ± 1.06% vs. 1.87% ± 0.79%, *p* < 0.05) (Figure 10(A3)).

Similarly, mice treated with PMS-HP (Figure 10(B2)) were associated with reduced plaque area in the region of the aortic root in comparison to the mice treated with PBS (Figure 10(B1)). As a result of using Image-Pro-Plus 6.0, mice treated with PMS-HP showed a significant reduction in the percentage of atherosclerotic plaque area in the region of the aortic root compared to mice treated with PBS, with statistically significant differences (33.3% ± 10.1% vs. 16.1% ± 3.69%, *p* < 0.05) (Figure 10(B3)).

Further, we performed HE staining on the tissue sections to assess the status of arterial plaques. In contrast to mice treated with PMS-HP (Figure 10(C2)), mice treated with PBS (Figure 10(C1)) showed an increased deposition of atherosclerotic plaques within the arterial lumen and a significant thinning of fibrous caps.

#### 3.3.5. PMS-HP Reduces Fibrosis in the Tricuspid Valve of the Heart

**Figure 11:**
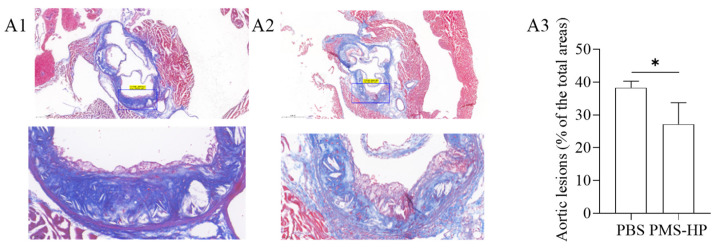
The images of collagen in aortic cross-section stained by Masson’s trichrome after different treatments. The images of collagen in aortic cross-section in mice treated with PBS (**A1**) or PMS-HP (**A2**); Comparisons (**A3**) of the collagen in aortic cross-section in mice treated with PBS and PMS-HP (n = 4, *p* = 0.017) (* *p* < 0.05).

According to Figure 11, the collagen content within plaques in the aortic root region was determined using Masson’s staining. In the control group treated with PBS, a significant amount of deep blue-stained collagen was observed within the plaques, indicating a high collagen content. Compared to the control group (Figure 11(A1)), mice treated with PMS-HP (Figure 11(A2)) displayed a lower collagen content within the plaques. Further statistical analysis of the data (as depicted in Figure 11(A3)) demonstrated that PMS-HP treatment effectively reduced the collagen content within the plaques (27.2% ± 6.55% vs. 38.3% ± 1.99%, *p* < 0.05).

#### 3.3.6. Biosafety Assessment

**Figure 12:**
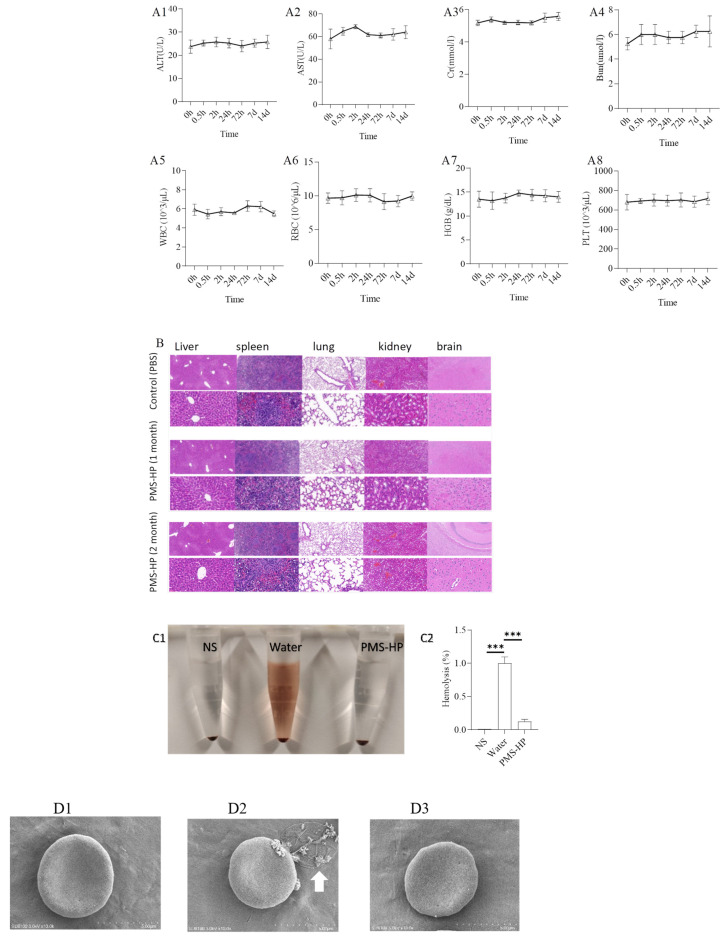
Safety and biocompatibility of PMS-HP in mice. Markers of blood were evaluated, including ALT (**A1**), AST (**A2**), Cr (**A3**), Bun (**A4**), WBC (**A5**), RBC (**A6**), HGB (**A7**), PLT (**A8**) (n = 4); Hematoxylin and eosin staining of the major organs (**B**), including the heart, liver, spleen, lung, and kidney; Hemolysis of different particles incubating with RBCs (n = 4, P1 = 0.003, P2 = 0.000). (**C1**) The picture for hemolysis with particles incubating with RBCs; (**C2**) Statistical results of hemolysis with particles incubating with RBCs (n = 3); (**D1**) SEM images of RBCs treated with NS; (**D2**) SEM images of RBCs treated with water added; (**D3**) SEM images of RBCs treated with PMS-HP. NS: normal saline (n = 3) (*** *p* < 0.001).

The evaluation involved the mice treated with PMS-HP. Blood samples were collected from the mice at different time points post-injection. The results, as shown in Figure 12 (A1–A8), indicated that PMS-HP did not exhibit significant toxicity.

To further examine the pathological condition of various organs following treatment for one month and two months, organ slices were subjected to HE staining. Figure 12B demonstrates that, compared to the control group treated with PBS, the major organs, including the heart, liver, spleen, lungs, and kidneys of mice in the PMS-HP treatment group did not exhibit significant pathological changes.

Furthermore, our study investigated whether PMS-HP caused hemolysis in RBCs. Co-incubation of PMS-HP with RBCs did not induce hemolysis (Figure 12(C1)). Absorbance measurements revealed that PMS-HP did not cause significant disruption or damage to RBCs when compared to water-induced hemolysis (Figure 12(C2)).

Moreover, SEM revealed that RBCs, when co-incubated with NS (Figure 12(D1)) or NS containing PMS-HP (Figure 12(D3)), exhibited a normal biconcave disc shape. In comparison, when RBCs were co-incubated with water (Figure 12(D2)), the concave shape of the cells disappeared, and the RBCs became swollen and ruptured (arrow in white).

### 3.4. Adsorption Mechanism

#### 3.4.1. Zeta Potential of the Particles

**Figure 13:**
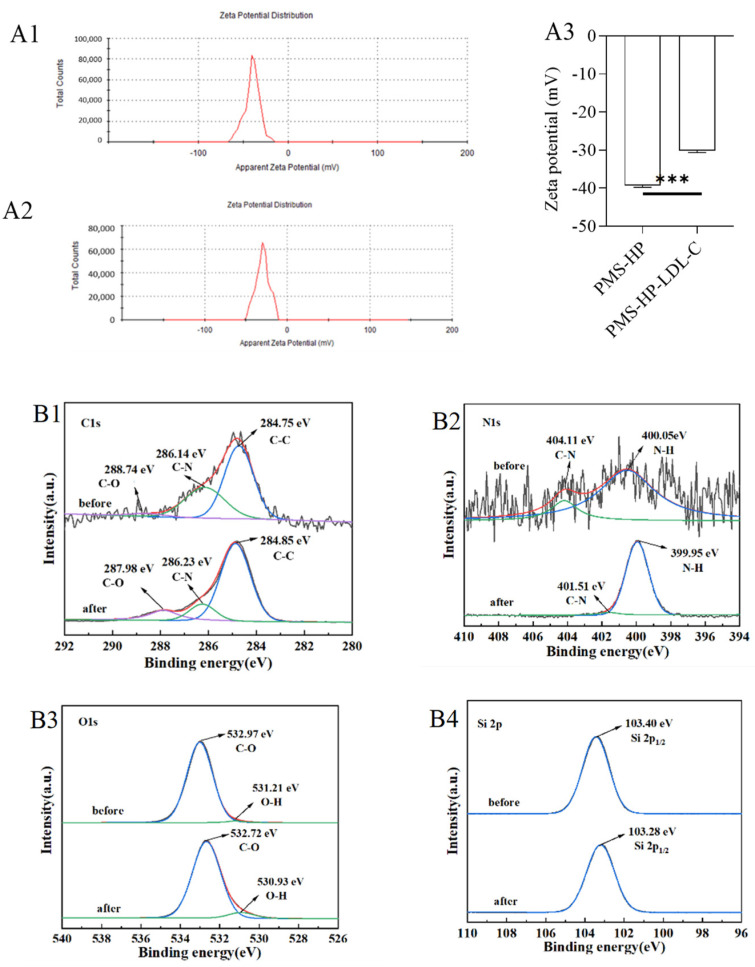
Adsorbing mechanism of PMS-HP. (**A1**): Zeta potential of PMS-HP; (**A2**): Zeta potential of PMS-HP after adsorbing LDL-C; (**A3**) Comparisons of the zeta potential among the different particles, including PMS-HP and PMS-HP after adsorbing LDL-C (n = 3, P = 0.000); C 1 s (**B1**), N 1 s (**B2**), O 1 s (**B3**), and Si 2p (**B4**) XPS spectra of PMS-HP before and after adsorbing LDL-C (n = 3) (*** *p* < 0.001).

In our study, we further investigated the mechanism of LDL-C adsorption by PMS-HP. As illustrated in Figure 13, in contrast to the initial state of PMS-HP (Figure 13(A1)), adsorbing LDL-C resulted in a significant increase in the potential level of PMS-HP-LDL-C (Figure 13(A2)). The observed difference was found to be statistically significant (Figure 13(A3)). Meaningfully, the electrostatic attraction played an important role in PMS-HP adsorbing LDL-C with a positive potential.

#### 3.4.2. XPS of the Particles

The chemical structure of PMS-HP was further investigated using XPS analysis, and potential interactions were evaluated during adsorption. PMS-HP samples were examined before and after adsorption to determine the presence of carbon (C), nitrogen (N), oxygen (O), and silicon (Si). As illustrated in Figure 13B, notable spectral changes were observed. As shown in Figure 13(B1), the C1s spectrum revealed three peaks at 284.75 eV, 286.14 eV, and 288.74 eV, corresponding to C–C, C–N, and C–O bonds, respectively. A distinct peak representing N–H groups was observed at 399.98 eV in the N1s spectrum (Figure 13(B2)) after LDL-C adsorption, indicating a noticeable change from pre-adsorption. In Figure 13(B3), the O1s spectrum exhibited primary peaks at 531.21 eV and 532.97 eV, which corresponded to O–H and C–O bonds, respectively, with significant changes observed in O–H bonding during lipid adsorption. The Si spectrum (Figure 13(B4)) showed no significant differences before and after adsorption.

### 3.5. Recovery and Recycling of PMS-HP

PMS-HP-LDL-C, subjected to high-concentration physiological saline washing, was able to achieve LDL-C release.

**Figure 14:**
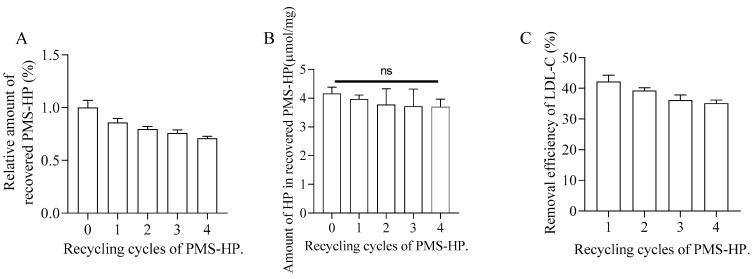
Recovery and recycling of PMS-HP. (**A**) The change in content of recovered PMS-HP; (**B**) The variation of HP in recovered PMS-HP; (**C**) The efficiency of LDL-C removal by recovered PMS-HP (n = 3).

As shown in Figure 14A,B related to quantitative analysis for recovered PMS-HP, most PMS-HP and HP on the PMS-HP were recovered and not lost. In addition, recovered PMS-HP consistently maintained a high level of LDL-C adsorption efficiency (Figure 14C).

## 4. Discussion

In this study, we investigated the potential of PMS-HP as a therapeutic agent for reducing blood lipid levels and preventing the progression of AS in ApoE^−/−^ mice. In our study, PMS-HP selectively reduced LDL levels significantly in vitro and vivo, contributing to a reduction in aortic AS severity in vivo. Moreover, we discovered that electrostatic attraction played a significant role in the mechanism of PMS-HP for adsorbing LDL-C in the blood. Additionally, based on our findings from XPS, we identified that N–H bonds and O–H bonds were crucial factors in the adsorption of LDL-C by PMS-HP.

With the aging of the global population and changes in lifestyle, AS has become one of the most common chronic diseases worldwide and a major cause of cardiovascular disease. Although there are many drugs available for the treatment of AS, their efficacy and adverse reactions still have limitations. Therefore, finding more effective treatment methods remains a hot research topic [29,30].

With respect to PMS reducing lipid concentration in vivo, it was demonstrated by Paul et al. that PMS could adsorb lipids in the gut and significantly reduce normalized rodent weight gain in Sprague–Dawley rats treated with a high-fat diet [31], which indicates the potential ability of PMS to reduce the levels of lipid. In addition, in the C57Bl/6 mice diet-induced obesity model, mesoporous silica particles supplemented in food successfully reduced adipose tissue formation (6.5 ± 0.5 vs. 9.4 ± 1.2 g), leptin levels (32.8 ± 7.4 vs. 16.9 ± 1.9 ng/mL), and 33% reduction of food efficiency in comparison to mice in the control group [32]. More importantly, based on the influence on lipid levels and lipid digestion products, mesoporous silica particles are emerging as an interesting option to act as a treatment for obesity and other metabolic conditions [33]. Similarly, in our study, we found that PMS could reduce LDL-C levels in the plasma in vitro.

However, PMS exhibited non-selective adsorption towards lipids, as shown in our study, contributing to reduced levels of HDL-C, proteins, and other components. In contrast, our functionalized PMS (PMS-HP) demonstrated selective adsorption of LDL-C, which did not have an influence on the levels of HDL-C and proteins in the blood.

Associated with safe ending and better histocompatibility, in comparison, it was confirmed in our study that PMS-HP in vivo showed no obvious toxicity to major organs and would not contribute to erythrocyte hemolysis, which contributed to the popular application in drug delivery [34,35].

Existing research has demonstrated that elevated blood lipid levels, particularly increased LDL-C levels, increase the burden of vascular plaques, accelerating the progression of AS. Moreover, elevated blood lipid levels result in an increased plaque burden, leading to arterial narrowing and ultimately causing plaque rupture, resulting in severe cardiovascular events and heightened mortality risk. Therefore, targeting the reduction of LDL-C would inhibit plaque progression and prevent major cardiovascular events such as MI, HF, and stroke, leading to a reduction in mortality rates [36]. Intravenous medications targeting the reduction of LDL-C were recommended to use proprotein convertase subtilisin/kexin type 9 (PCSK9) inhibitors, which could interfere with the binding of PCSK9 to LDL receptors [37]. However, existing research has indicated that PCSK9 inhibitors could lead to various adverse reactions, including allergic reactions, immune antibody generation, neurocognitive events, musculoskeletal issues, and abnormalities in blood sugar [38,39]. In extracorporeal applications aimed at reducing LDL-C through lipoprotein apheresis, materials such as resins were used. Despite lowering LDL-C levels to a certain extent, its systemic application was greatly restricted [40]. In comparison, PMS-HP possesses several advantages. Firstly, PMS-HP acted directly on LDL-C and was widely recognized for its significant role in the development of AS. Secondly, its action relied on an adsorption mechanism, thereby minimizing potential toxic effects. Moreover, PMS-HP held value in extracorporeal applications, serving as a vital component for lipid adsorption in extracorporeal lipid adsorption and potentially as an integral part of dialysis machines. Most importantly, the adsorbed LDL-C could be released, facilitating its re-circulation.

LDL-C is composed of TG, cholesterol esters (CE), and unesterified cholesterol (UC). Its surface is primarily composed of ApoB-100, which has the ability to bind to heparin, resulting in the precipitation of LDL-C [41]. Building upon the foundation of heparin, Li et al. developed a magnetic nanocomposite material by conjugating heparin with chitosan. This innovative approach effectively facilitated the removal of LDL-C from plasma in vitro [42]. This result was consistent with our PMS-HP selectively removing LDL-C from the plasma in vitro.

Several factors were identified that potentially contributed to the effect of PMS-HP in reducing LDL-C and preventing the progression of AS. Firstly, PMS-HP acted as a solid-phase extraction material to adsorb LDL-C, resulting in a reduction in LDL-C. As was shown in our study, LDL-C adsorbed by PMS-HP could be released with a high-concentration sodium chloride solution, proving the fact that PMS-HP could adsorb LDL-C through physical sequestration. Secondly, the formation of PMS-HP-LDL-C complexes resulted in a disruption of the normal interaction between blood lipids and lipase, resulting in impaired lipase activity [33].

In terms of the possible mechanism by which PMS-HP adsorbed LDL-C, as demonstrated in our study, PMS-HP’s negative potential level provides more opportunities for PMS-HP to capture LDL-C with a positive charge. As a result, PMS-HP adsorbs LDL-C through the electrostatic interaction, which is similar to PMS [43,44]. In addition, it was found that N–H and O–H played an important role in PMS adsorbing substances during the process [45]. Similar to our findings, N–H and O–H played an important role during the process on the basis of the result of XPS.

With respect to N–H bonds, nitrogen atoms in PMS-HP possess lone pair electrons, which could form hydrogen bonds with polar groups, such as the oxygen atoms in LDL-C. This aids in the binding and adsorption of LDL-C. With respect to O–H bonds, oxygen atoms in PMS-HP could engage in hydrogen bonding with polar groups in LDL-C. These oxygen atoms could donate lone pair electrons, thereby forming hydrogen bonds with LDL-C, promoting their adsorption and binding. Electrostatic interactions occurred between regions of positive and negative charges within molecules. In addition, the O–H groups in PMS-HP could serve as hydrogen bond acceptors and interact electro-statically with hydrogen atoms or other positively charged regions in LDL-C. Furthermore, zeta potential measurements were conducted, revealing that following the adsorption of LDL-C by PMS-HP, the negative zeta potential decreased [45].

Moreover, silicon was important for preventing AS. It was found that silicon-enriched pork improved lipoprotein profiles, decreased VLDL-C oxidation, and upregulated the expression of LDL-C receptor genes in aged rats fed an atherogenic diet, suggesting that it could be beneficial in the prevention of AS [46]. Meaningfully, silicon in the PMS-HP might influence the progression of AS.

In our study, the fluorescence intensity for the brain was taken as a reference. Although it seemed that there was an increasing trend in the number of signals for the brain and heart, PMS-HP did not pass through the BBB and accumulate on the vascular wall. The following are several possible explanations: In theory, the BBB is a semi-permeable barrier where wedge-shaped endothelial cells are arranged within capillaries, forming extensive tight connections. The BBB can exclude over 98% of small-molecule drugs and all large-molecule therapeutic agents from entering the brain. Moreover, it allows the passage of lipid-soluble substances while preventing the passage of water-soluble ones [47]. PMS-HP is water-soluble with a particle size of approximately 150 nm. Next, in our experiments, we utilized nitric acid digestion of brain tissue to detect the level of silicon elements within. The experimental results revealed no significant presence of silicon elements, suggesting that PMS-HP did not pass through the BBB. Moreover, in our experiments validating the in vivo safety of PMS-HP, no notable brain tissue damage was observed. Taking all these factors into account, it is evident that PMS-HP did not breach the BBB. Therefore, the utilization of brain tissue fluorescence intensity as a reference group was justified. Variations in brain tissue fluorescence might have stemmed from differences in the surface moisture content of the brain tissue.

However, there are also limitations in our study. (1) The fluorescence of PMS-HP-Cy5 exhibited quenching properties. Although we did not detect fluorescence in the major organs during the IVIS imaging on the 14th day, it does not guarantee the complete elimination of nanoparticles from these major organs. Furthermore, we only assessed the major organs, and we cannot rule out the possibility of nanoparticle accumulation in other organs. (2) Regarding the specific dose–response relationship of nanoparticles in the body, given the difficulty of purifying and quantifying the absolute mass of individual nanoparticles from the body, we did not accurately determine the amount of LDL-C that 1 mg of nanoparticles could adsorb within the body.

Based on our research, we believe that PMS-HP can effectively adsorb LDL-C both in vitro and in vivo. For future studies, we propose exploring two key areas. Firstly, in the in vitro research domain, constructing an extracorporeal lipoprotein apheresis system centered on PMS-HP to further assess its efficacy in removing LDL-C. Secondly, within the realm of in vivo research, considering further evaluations of PMS-HP’s application in large animal models and potentially in clinical settings to better elucidate its anti-atherosclerotic effects.

## 5. Conclusions

In our research, we made a discovery in the field of LDL-C lowering medications by developing a novel drug that harnessed the principles of charge attraction and functional group interactions to exhibit selective adsorption of LDL-C, contributing to reduced intravascular plaque formation and inhibited vascular remodeling.

## Figures and Tables

**Table 1:** Experiment of standard curve.

Number of Tube	1	2	3	4	5	6	7
TB solution, mL	0.5	0.5	0.5	0.5	0.5	0.5	0.5
Heparin sodium, µL	0	40	80	100	160	200	240
Water, µL	500	460	420	400	340	300	260
Hexyl hydride, mL	1	1	1	1	1	1	1
Concentration of heparin sodium, µg/mL	0	10	20	25	40	50	60

## Data Availability

The authors declare that all the data in this manuscript are available upon request.
